# Health professional perceptions regarding screening tools for developmental surveillance for children in a multicultural part of Sydney, Australia

**DOI:** 10.1186/s12875-018-0728-3

**Published:** 2018-04-02

**Authors:** Pankaj Garg, My Trinh Ha, John Eastwood, Susan Harvey, Sue Woolfenden, Elisabeth Murphy, Cheryl Dissanayake, Katrina Williams, Bin Jalaludin, Anne McKenzie, Stewart Einfeld, Natalie Silove, Kate Short, Valsamma Eapen

**Affiliations:** 1 Department of Community Paediatrics, Liverpool Hospital, Liverpool, NSW Australia; 2 School of Women’s and Children’s Health, UNSW, Sydney, Australia; 3 School of Medicine, University of Sydney, Sydney, Australia; 4 Specialist Disability Health Team, Fairfield, Australia; 5 Ingham Institute of Applied Medicine, Liverpool, Australia; 6 ICAMHS, South Western Sydney Local Health District, Sydney, NSW Australia; 7 University of Western Sydney, Sydney, Australia; 8 School of Public Health, University of Sydney, Sydney, NSW Australia; 9 Ingham Institute of Applied Medicine, Liverpool, NSW Australia; 10 School of Medicine, Griffith University, Gold Coast, QLD Australia; 11 Department of Community Paediatrics, Sydney Local Health District, Croydon Community Health Centre, Croydon, Sydney, Australia; 12 School of Nursing and Midwifery, Griffiths University, Brisbane, QLD Australia; 13 Sydney Children’s Hospital Network (Randwick), Randwick, Australia; 14 UNSW, Sydney, Australia; 15 NSW Ministry of Health, Sydney, Australia; 16 Olga Tennison Autism Research Centre, La Trobe University, Melbourne, VIC Australia; 17 Royal Children’s Hospital, Melbourne, VIC Australia; 18 University of Melbourne, Melbourne, Australia; 19 Murdoch Childrens Research Institute, Melbourne, Australia; 20 South Western Sydney Local Health District, Sydney, NSW Australia; 21 Child and Family Health Nursing, Primary and Community Health, South Western Sydney Local Health District, Sydney, NSW Australia; 22 Centre for Disability Research and Policy and Brain and Mind Centre, University of Sydney, Sydney, Australia; 23 The Children’s Hospital at Westmead, Westmead, NSW Australia; 24 University of Sydney, Sydney, Australia; 25 Liverpool Hospital, South Western Sydney LHD, Liverpool, NSW Australia; 26 UNSW, ICAMHS, South Western Sydney LHD, Sydney, NSW Australia; 27 Chair, Infant Child and Adolescent Psychiatry, University of New South Wales, Head, Academic Unit of Child Psychiatry, South West Sydney (AUCS), ICAMHS, Mental Health Centre, L1, Liverpool Hospital, Elizabeth Street, Liverpool, Sydney, NSW 2170 Australia

**Keywords:** Developmental screening, Perceptions, Professionals, PEDS, ASQs, Child development

## Abstract

**Background:**

Encouraging early child development and the early identification of developmental difficulties is a priority. The Ministry of Health in the Australian State of New South Wales (NSW), has recommended a program of developmental surveillance using validated screening questionnaires, namely, the Parents’ Evaluation of Development Status (PEDS) and Ages and Stages Questionnaire (ASQs), however, the use of these tools has remained sub-optimal. A longitudinal prospective birth cohort “Watch Me grow” study was carried out in the South Western Sydney (SW) region of NSW to ascertain the uptake as well as the strategies and the resources required to maximise engagement in the surveillance program. This paper reports on a qualitative component of the study examining the attitudes, enablers and barriers to the current developmental surveillance practices, with reference to screening tools, amongst health professionals.

**Methods:**

Qualitative data from 37 primary health care providers in a region of relative disadvantage in Sydney was analysed.

**Results:**

The major themes that emerged from the data were the “difficulties/problems” and “positives/benefits” of surveillance in general, and “specificity” of the tools which were employed. Barriers of time, tool awareness, knowledge and access of referral pathways, and services were important for the physician providers, while the choice of screening tools and access to these tools in other languages were raised as important issues by Child and Family Health Nurses (CFHN). The use of these tools by health professionals was also influenced by what the professionals perceived as the parents’ understanding of their child’s development. While the PEDS and ASQs was utilised by CFHNs, both General Practitioners (GPs) and paediatricians commented that they lacked awareness of developmental screening tools and highlighted further training needs.

**Conclusions:**

The results highlight the practical challenges to, and limited knowledge and uptake of, the use of recommended screening tools as part of developmental surveillance. There is a need for further research regarding the most effective integrated models of care which will allow for a better collaboration between parents and service providers and improve information sharing between different professionals such as CFHNs GPs, Practices nurses and Paediatricians involved in screening and surveillance programs.

## Background

An Australian Early Development Census (AEDC) has revealed that 22% of children are developmentally vulnerable in one or more domains in their first year of formal schooling, with the potential to negatively impact on their long-term capacity to learn [1]. This situation is not confined to Australia and is of particular concern due to the known importance of the early years for child development [2]. Internationally, and in Australia, developmental surveillance programs have been instituted as a means of early identification, by including the use of validated screening tools, as well as a focus on prevention through child health promotion activities [3, 4].

### Developmental screening and surveillance

The prevalence of parental concerns of their child’s development using the Parents’ Evaluation of Development Status (PEDS) is about 13.8% for ‘high developmental risk’ and 19.8% for ‘moderate developmental risk’ [5–9]. Therefore, robust processes are needed to identify children with developmental delays [10]. Developmental screening and surveillance are important population-based approach for the early identification of developmental problems, and the use of developmental screening tools allows parents to discuss questions about their child’s development which provides a clear guidance to health professionals about decisions for monitoring or referral. Developmental surveillance involves longitudinal elicitation of parental concerns, obtaining an informed developmental history, performing skilled observations of children, and soliciting information from child care providers when concerns regarding a child’s development become evident.

The screening component of surveillance refers to the periodic administration of standardised tools to enhance early detection and improved health and learning outcomes [11]. A number of developmental screening tools, either parent-completed or administered by a health professional, have been validated, and can facilitate the process of early identification on the pathway to early intervention [12]. Despite recommendations for the use of screening tools such as Parents’ Evaluation of Development Status (PEDS) and the Ages and Stages Questionnaires, (Age and Stages Questionnaire; Ages and Stages Questionnaire: Social Emotional, ASQs), their use by health professionals has generally remained sub-optimal globally [13–15]. On the other hand, the implementation of the use of routine screening tools by paediatricians have been shown to quadruple the referrals for developmental delays [16]. Similarly studies have shown that the implementation of the American Academy of Paediatrics (AAP) guidelines for developmental screening in 17 paediatric practices resulted in up to 85% of eligible children being screened for developmental problems [17].

#### Health service context for developmental screening

The best approach for developmental screening varies according to the local context within national health systems, and a ‘one-size- fits-all’ approach is debatable [18]. The United Kingdom (UK) child health surveillance program has evolved into a child health promotion program, with lesser number of ‘universal’ child health visits compared to the AAP guidelines [3, 4], while New Zealand uses a defined well-child care program of regular health visits, delivered through the Plunket health nurses [19]. In North America a variety of innovative approaches are being discussed and explored for delivering child health promotion such as the use of non-health professional providers [20].

In the Australian state of New South Wales (NSW) parents are provided the Personal Health Record (PHR, commonly known as the ‘Blue Book’) at their child’s birth, with information provided about the recommended developmental checks. Parents are linked to child and family health nursing services in their local area and information about developmental screening is provided to parents at the time of universal health home visit (UHHV) and/or at the time of visit to an early childhood health clinic [21]. Parents from culturally and linguistically diverse (CALD) backgrounds residing in the Local Health District are more likely to attend GPs for their child’s health checks [22]. However, it has been shown that at times parents’ concerns are not recognised or acknowledged and there may be gaps in sharing health information at the time of health visits [22, 23].

A large population based study of general practice visits in Australia has demonstrated that for every 100 visits to GPs, four visits are made for immunisation for pre-school children [24]. Hence, GPs are in a unique position to deliver developmental screening within child surveillance activities, especially given that parents from CALD backgrounds seek  their services in preference to Child and Family Health Nurses (CFHNs) services [22, 23]. GPs conduct developmental screening opportunistically as universal child development screening activities are ‘optional’, and do not attract any specific funding or incentives. In contrast, GPs are provided with incentive payments through Medicare (government-funded health insurance for all Australian residents and citizens) for following up unvaccinated children [25, 26]. Another approach in the recent past (2008–2015) to encourage screening by Australian GPs, and funded through Medicare, was a national ‘one-off’ Healthy Kid’s Check (HKC) for 4 year old children. The HKC focused on a number of domains of well-child visits including developmental screening [27]. However, this program was ceased due to a variety of factors including a slow initial uptake by GPs (with subsequent increase over the 7 years of the program), and emerging evidence that components of the HKC were not evidence-based. In addition, the age at the time of the screening for HKC was debatable. It is speculated that the discontinuation of HKC will result in missed opportunities for developmental screening for children [27–29].

#### Screening tools used in NSW

The PEDS is a 10 − item parent-completed standardised questionnaire, which is used to elicit parental concerns around child development as a primary tool [30]. It is included in the PHRs in NSW to be completed at six, 12 and 18 months and at two, three and 4 years of age. Parents are encouraged to complete the PEDS in the Blue Book and discuss any concerns with the CFHNs or GPs during health checks or immunisation visits.

In NSW, the ASQs are most often used as secondary developmental screening tools as the next step for more in-depth exploration of developmental concerns raised after  completion of the PEDS. The ASQs comprises 19 age appropriate questionnaires (4-months to 5-years of age) and the ASQs: Social-emotional has eight questionnaires (6-months to 5-years of age). The questionnaires are written at a 4th -5th primary school reading level and ‘cut-off’ scores and pathways provide health professionals with direction regarding continued routine screening, monitoring and review, and/or direct referral for assessment [31]. The ASQs are also used as an initial screening tool for children with existing identified vulnerabilities, such as families enrolled for a sustained home visiting program [31]. Healthcare interpreters are utilised by NSW Health services and can be booked for appointments with CFHNs or telephone interpreter services can be used by GPs. Table 1 summarises the characteristics of the ASQs and PEDS as screening tools [13, 32, 33].

**Table 1:** Comparison of PEDS and ASQs as developmental screening tools

Characteristic	PEDS	ASQ
Screening approach	Parents' developmental concerns	Parents provide information about child’s skills
Format	10 questions covering 9 developmental concerns, 1 page Response options: no/yes/a little	30 questions covering 5 developmental domains, 3 pages Response options: yes/sometimes/not yet
Example of item	Expressive language: “Do you have any concerns about how your child talks and makes speech sounds?”	Communication skill at 18 mo: “Does your child say 8 or more words in addition to ‘Mama’ and ‘Dada’?”
Time to screen	5 min of parent time	10–15 min of parent time
	1–2 min for provider/staff to score	1–2 min for provider/staff to score
Scoring summary	Yields overall pass/fail score Path A: 2 significant concerns (refer for evaluation) Path B: 1 significant concern (administer formal skill-based screen)	Yields overall pass/fail score Each of 5 domain subscales (eg, communication, fine motor) yields pass/fail score
Sensitivity	0.74–0.79 (moderate)	0.70–0.90 (moderate to high)
Specificity	0.70–0.80 (moderate)	0.76–0.91 (moderate to high)
Available languages	Arabic, Chinese, Dinka, Vietnamese, Korean, Lao, Somali, Khmer, Tamil, Thai, Hindi, Indonesian	Arabic, Spanish, Chinese, Korean

#### Training and systems used by professionals for developmental screening tools

The use of the screening tools by health professionals is related to the training they have received in these tools. In Australia CFHNs have training in the use of PEDS and ASQs, including scoring and interpretation of results as a core component of their professional practice. In addition, anticipatory guidance and the provision of educational material on child development is provided for parents as a normal part of health promotion. The screening tools are routinely used, and outcomes documented in the child’s medical record, including a plan of action for review or referral to GPs and other services as needed. Medical records of the CFHNs are subject to file audits for quality improvement purposes including adherence to recommended pathways [34, 35]. But the population level coverage of developmental screening has remained limited in many Australian states, including New South Wales (NSW) where only about 50% of children between 0 to11 months, and 35% of children between 1 to 4 years of age access child and family health nursing services [36, 37].

Training for GPs in Australia includes online modules linked to written guidelines for preventive developmental activities for use in general practice (commonly known as the ‘red book’) [38]. The focus of this training is to provide GPs with a broad knowledge and a preventative approach to healthy eating, behaviour management, child development, and hearing and vision checks. GPs are also encouraged to consider the use of developmental screening tools such as PEDS, or an alternative approach of ‘red flags’ for developmental milestones is suggested as a means of identifying developmental delays to ensure early referral [38]. A study from general practices in Queensland is reassuring and has demonstrated that mostly GPs were diligent in identifying children with developmental concerns during routine health checks [28].

The training for paediatricians in Australia includes a mandatory term in community, developmental and behavioural paediatrics in order to provide them with the knowledge and practical experience of developmental screening tools [39]. A clinical report from paediatricians as to their strategies for identifying children with developmental delays, diagnostic formulation and further management plan including referrals and monitoring pathways is a standard practice in Australia. But the challenges for GPs and paediatricians include the waiting lists for diagnostic developmental services when children with delays are identified, and sometimes limited access to allied health professionals for early intervention [40].

### Research context

The findings reported in this paper are one part of the qualitative component of the ‘Watch Me Grow’ (WMG) study. The WMG longitudinal birth cohort study was established in the South Western (SW) Sydney region with the broad aim of generating robust evidence to inform policy makers and service providers on maximizing the uptake of the developmental surveillance program [41]. The description of the study cohort and quantitative evidence assessing the risk factors and prevalence of parental developmental concerns for their children has been reported elsewhere [42–44]. We have also recently reported the findings from the qualitative component of the study on the parent’s experiences for accessing services for their children’s health and development [22].

### Aims

As highlighted above in section 1.1, prior research have highlighted that the health professionals find it a challenge to use screening tools for identifying developmental delays. Therefore, further knowledge about health professionals perceptions around use of developmental screening tools in a multicultural area are of interest and have the potential to inform clinical practice and policy development. The qualitative component of the WMG study was planned with an aim for providing an in-depth exploration and context of the use of screening tools by health professionals, particularly if there were any differences in the way between physicians (GPs and paediatricians) and CFHNs use of screening tools for developmental surveillance. The overall question which needed to be answered was why do some children get developmental screening and get identified early, and what are the main barriers and enablers from the perspectives of the resources, capacity and training needs of professionals to encourage more frequent use of screening tools by them. The other question was to answer how best to facilitate and encourage the regular attendance of children and parents for routine health and developmental checks.

## Methods

### Ethics

The study was approved by the Human Research Ethics Committees (HREC) of South Western Sydney Local Health District (SWSLHD) and the University Of New South Wales (UNSW).

### Setting

The SW Sydney area ranks low on the Index of Relative Socio-economic Disadvantage, a measure of relative disadvantage in terms of education, employment, income and occupation [45]. The population is relatively young with about 15% of residents being children 0 to 8 years of age [46]. The region has a large population of CALD persons with approximately 34% of residents born overseas (some suburban areas up to 50%), and about 35% of the population reporting English as their second language (up to 70% in some suburban areas). Other than English, the main languages spoken in the area include Arabic, Chinese, Vietnamese, Khmer, Korean, Greek, Spanish, Italian and Serbian. Unemployment rates are high in the area (5.2 to 22.3% as compared to the NSW average of 4.7%), and median individual incomes for over a third of the population are significantly less than the state average [34]. Also, there are many areas in the region with a very high child social exclusion risk using income and non-income measures of children’s risk of social exclusion [47].

The NSW Ministry of Health provides recommendations for a schedule of well-child visits to primary health care professionals in the area using the PHRs for families with young children [48]. Child health promotion activities are a top priority in this region, with a focus on availability of universal services and development of targeted services for specific vulnerable groups [49]. Since the conduct of the WMG study, relevant parts of the PHR including the PEDS, translated to other languages, are actively promoted [47]. Similarly translations of other educational programs for promoting child development literacy such as the ‘Love, Talk, Sing, Read, Play’ have been made available [50]. In addition, there is an improved access to supported playgroups, and a number of publicly funded outreach community paediatric clinics for early identification of developmental problems [51]. A perinatal coordination program, for early identification of vulnerable and at risk families at the time of birth and a sustained home visiting programs for these families are also active in the region [52]. There are dedicated interpreter services not only for face to face interpreting but also for video and telephone interpreting [52].

### Recruitment and sampling

Qualitative data was collected from a total of 37 health professionals within the study area using a purposeful sampling strategy [53]. Purposeful recruitment began by first identifying experts in the field and those working in the authors’ departments. Subsequently snow-balling, opportunistic, and convenience sampling strategies were used to identify the participants [53].

Information about the study was presented to potential participants using participant information sheets and written informed consent was obtained which included consent for audio-recording. Two researchers, a male Community paediatrician and a female CFHN with experience in conducting focus groups and in-depth interviews collected the data. One of the researchers worked within the Local Health District and some of the participants were colleagues to both of them. Demographic data from health professionals were collected on a standardised form. Qualitative data were collected at the workplace of participants. The focus groups lasted 60–90 min while individual interviews lasted 15–45 min.

### Data collection measures

A semi-structured interview guide with five broad open ended questions was used by the researchers to initiate and guide data collection for both individual interviews as well as the focus groups (Table 2). The questions focused on elaborating responses to an opening question such as, why only some children receive developmental screening, thereby, resulting in only some children of developmental delays being identified early. The other questions explored the usefulness of the Blue Book (PEDS) for developmental screening and surveillance. The questions also explored the factors which were likely to encourage or discourage developmental surveillance using screening tools, and the educational and training needs of health professionals in screening tools (Table 2).

**Table 2:** Initial Interview Guide

Question No^a^	Question
1	Why do some children receive developmental screening/surveillance and therefore have developmental concerns picked up early?
2	Why do some children miss developmental screening/surveillance?
3	Is the Blue Book^$^ useful for screening/surveillance?
4	If Not, why is the Blue Book not useful?
5	What would encourage universal developmental surveillance using screening tools?
6^b^	Any likely factors which encourage or discourage developmental surveillance using screening tools?
7^b^	What are the training needs of health professionals?

^a^ Most of the time a single broad question on the participants’ views on developmental surveillance was an adequate prompt to gain data in line with the questions, ^b^Q 6 and 7 were occasionally used as a probing question, $ Participants  were directed to PEDS screening tool in the Blue Book if participants did not mention this

In-depth interviews allowed for the exploration of individual experiences and perceptions in greater detail, while the focus group with the CFHNs allowed discussion between participants, prompting further recollection of individual experiences by guided interactions among experienced and relatively new graduate nurses. It was logistically easier to undertake in-depth interviewers with GPs and paediatricians to fit in with their individual practice schedules and to conduct focus groups for CFHNs at a Community Health Centers after they had returned from home visits or clinics in the community.

Field notes were made and recorded data was transcribed verbatim by a professional service. A sub-section of the transcribed data was verified for accuracy by the first author. Data was exported and analysed using NVivo Qualitative software [54].

### Data analysis

In keeping with the qualitative data analysis tradition [55], the coding process started with data collection and involved identifying and exploring recurring words, themes and concepts and then grouping them into categories using a process of constant comparative analysis. This coding process used the “word frequency” and “text search” query commands of the software [52], and evolved from open to axial coding by subsuming sub-categories (“child nodes”) under the major categories (parent nodes). Individual participant nodes were created incorporating the demographic characteristics of participants using the ‘Nodes classifications’ option of the software. This enabled the exploration of data using attributes such as the type of profession, age-group and gender using “group query”, “coding” and “matrix coding” commands. The data analysis did not differ between focus groups and individual interviews.

Data analysis ceased when thematic saturation was achieved. The negative case (i.e. where the participant raised views different from others) was acknowledged. An example of this was a Paediatrician with a more intensive training in child developmental- behavioural paediatrics reported using screening tools in practice, while the majority did not.

Two researchers coded the data independently, one of them with no participation in data collection. An inter-rater reliability coefficient of Kappa = 0.49 (percentage agreement: 94.25%) was achieved. When there were differences in the interpretation of data or codes, discussions were held with team members until agreement was reached. The preliminary findings were subjected to multi-source feedback which informed further data analysis [56]. It was not possible to gather feedback from everyone who participated due to time and resource constraints.

## Results

Thirty-six of the 37 participants gave permission for their interview/focus group to be audio recorded. One participant did not agree to be interviewed face to face; and instead the consent for using the written responses to interview questions by email was provided for analysis. Individuals interviewed in the current study included three nurse managers, one Out-of-Home Care coordinator (social worker), two GP practice nurses, five CFHNs, six GPs, seven paediatricians and one senior child health medical officer. Twelve CFHNs (different from the five individually interviewed) participated in two focus groups. The demographic characteristics of the participants are shown in Table 3.

**Table 3:** Characteristics of the participants

Data collection procedure	Number (n)	Median, years(IQR)	Median Years of experience(IQR)	Gender	Roles
Individual Interviews	25	53(44–58)	20(14–28)	Females-16	Nurses-10
				Males-9	Allied health-1
					Paediatricians-8
					GPs-6
Focus group 1	8	38 (32–43)	7 (0.5–18)	All females	All nurses
Focus group 2	4	49 (41–54)	15 (12–21)	All females	All nurses

*IQR* Interquartile range

Two major categories emerged from the data: ‘difficulties/problems’ relating to developmental screening tools for developmental surveillance programs, and ‘positives and benefits’ related to using these tools for assessment of children. The main themes which emerged within the ‘difficulties/problems’ were ‘parents not bringing their children for health visits’, professionals citing lack of awareness and knowledge of these tools, a perception of problems of parental understanding of their child’s development, reliance on clinical judgement for identifying developmental problems, professionals’ views on the specificity of the tools, and the health system related barriers. The ‘positive and benefits’ included the professionals’ views on the benefits of these tools and an acknowledgement of being able to evaluate parental concerns in a standardised way when these tools were used.

### ‘Difficulties/problems’

The health professionals (CFHN’s, GP’s and paediatricians) described various barriers and limitations to conducting developmental screening using PEDS and ASQs screening tools. These barriers included factors that impacted parents accessing routine health checks such as transport issues, the lack of flexibility of the clinic timings, and professional level barriers such as the awareness and knowledge of screening tools.

#### Perceived barriers for parents to access services: ‘Parents don’t bring their child routinely for health checks’

Factors that impacted on parents accessing developmental surveillance services were a major theme that arose from the data – all health care professionals highlighted similar views.

##### ‘Transport issues’

CFHNs spoke about various factors that had an impact on parents’ willingness/ability to access and/or attend services, ‘*We are expecting the client to come to us, so often they don’t*’ and ‘*it could be a lack of transport*’.

##### ‘Language’

CFHNs also highlighted language as a barrier for participation in developmental screening programs, ‘*PEDS is only in English* [often not using PEDS in other languages]*, think that probably affects* [access to developmental screening]’. Paediatricians also indicated that language may pose a barrier to attendance: *“…language is another barrier…if you don’t speak the same language; I think it’s difficult to explain to the parents and sometimes they don’t understand...” (Paediatrician).*

##### ‘Parents ‘awareness of the schedule of recommended visits in the ‘Blue Book’ (PHR)’

Paediatricians also spoke about access for screening and that ‘*the Blue Book very often isn’t used, families lose it, or forget to bring it’*.

CFHNs perceived that parents mostly used the PHR as a record of their child’s immunisation, with some parents also aware of the developmental screening schedule and the importance of developmental surveillance.

##### ‘Parents’ choices for engagement in developmental screening’

CFHNs indicated that parents’ engagement with services was related to a number of issues. Firstly, ‘*the voluntary nature of the surveillance program*’ was perceived to have an impact on whether or not parents chose to attend services for the recommended schedule of child development checks. This may also be related to a lack of knowledge about health services in the community and the impact of parents returning to work. One CFHN stated, ‘*families who are quite isolated, who don’t have connections in the community, who don’t know that there is an early childhood health nurse they can go to, and often the parents are working’*. CFHNs noted that parents may find it difficult to attend for developmental screening due to the ‘*inflexibility of services*’ in reference to the need for an appointment rather than parents also having the option of attending a drop-in clinic.

##### ‘Parents’ understanding/beliefs of the need for health and developmental screening checks’

Health professionals in this study believed that the parents’ understanding of normal child development may influence how parents respond to questions in the PEDS,

*‘…….at a six month assessment the child, they will (parents) say no (they have no concerns), but when you check the child is not doing this [activity within normal development parameters] and then do a more complex screening assessment…’,(CFHN).*

In this instance, differences in parents and professional knowledge about child development may become evident. CFHNs considered the opportunity for further discussion with the parent and the use of ASQs could be potentially missed if the parent had stated, ‘No concerns’ and additional exploratory questions were not used.

GP’s did not highlight attendance to them as an issue for developmental screening and surveillance, but rather noted that parents attended at the recommended times primarily for immunisation.

#### Perceived barriers for professionals’ use of recommended developmental screening tools

##### ‘Knowledge of screening tools’

GPs and paediatricians highlighted that they had limited knowledge of the PEDS and ASQs, and suggested that training for GP’s would be necessary to not only increase knowledge but also awareness:

*“…It would be good if…someone taught us how to do that, how to look at this properly” (GP).*

GPs acknowledged that they didn’t use these tools in the Blue Book: *“Blue Book? Most of the time we don’t refer to that” (GP).*

##### ‘Specificity of screening tool’

Professionals particularly the CFHNs raised concerns regarding the use of PEDS and would prefer to check specific milestones to lead into a discussion with parents. Although the PEDS: Developmental Milestones tool is in existence, CFHNs did not mention its use. This may be because other resources (such as those linked to the ASQs for promoting development) were utilised more often by CFHNs, and because of licencing issues related to PEDS: DM.

‘*I think it* [PEDS] *needs to have more specific developmental information of what are positives, what you could do, how you could play. Eighteen months, learn to kick a ball....if you put developmental phases and times and about How this should happen and this is how you help it to happen, it might get parents more engaged’.*

##### ‘Context of practice and health system factors’

Health professionals identified some of the contextual barriers to actively and routinely completing developmental screening measures,

*‘I think, that the GPs have to be interested in children to do it* [developmental screening] and ‘*if the doctor’s by himself, (in a)small group practice (or) solo practice, and there’s no practice nurse around, they have no time to do it, especially in (the) winter time with a lot of cough and colds’* (GP).

Some other broader health systems barriers such as the continuity of the professional service provider, *‘We do not see the same child, you know, over and over again. We do not see, the significance of (development progress) in the child’*[provided by a ‘one-off’ assessment in the context of a broader knowledge of the child’s development]*,* and Medicare funding systems *‘I don't see using screening tools for assessments are something that Medicare's happy with GPs doing and charging* [longer duration consultations]’*(GP).*

CFHNs discussed their practice specific suggestions for more frequent use of screening tools. In particular they suggested that despite the presence of a centralised appointment system changes were needed in the way appointments were offered to the families, and there may still be value in ‘drop-in’ clinics. They also highlighted the barriers of time and need for another appointment for some children when delays were identified on a primary screening tool.

Some health professional managers acknowledged that use of a ‘*one-stop shop* ‘may encourage an increased use of routine developmental screening in the SW region. They suggested greater collaboration between CFHNs, GPs and other services could occur with co-location and also provide the benefit of improve access for parents.

##### ‘Reliance on clinical judgement’

Paediatricians indicated that they often relied on their knowledge and experience of developmental milestones (reliance on clinical judgement) to identify concerns (rather than relying on screening tools).

it was also highlighted by paediatricians that although they may not use any specific screening tool, they play with the child to assess different developmental domains and identify delays using this clinical approach:

*‘I basically ask developmental question to the parents based on normal development.... and, their learning and gross skills and if I find delay then I do the screening test in my room using the basic tools (toys and equipment) I have available like cubes, crayons, books, colours, draw a man ....depending on whatever I have available’.*

Similar approaches were used by GPs who often based their assessment on clinical observations, ‘*lot of* [where a child is with regards to their development] *is based on observations and this does not get recorded* [in the Blue Book]’ (GP).

### Positives and benefits

#### ‘The importance of screening tools’

Despite the perceived limitations of the screening tools, many of the health professionals, particularly the more recently graduated CFHNs highlighted the benefits of available screening tools for identifying concerns regarding children’s development.

‘*I think generally I find the clients find it* [screening tools, PEDS and ASQs] *useful, the staff find it useful. At least it gives you – and the parents, a guide to go by, what things to come in for that visit to talk about’ (CFHN).*

It helped the professionals in their the first step of initiating a conversation with the parents, ‘*the PEDS, is useful as a first step to then cue the person who should be looking at the PEDS to go, oh I need to ask more questions….it’s only a step on to then use the ASQs ora, more complex screening assessment’.*

The following quote is any example of the overall response of professionals to the use of developmental screening tools as a part of developmental surveillance: *“…I think it’s important and I don’t think it’s done enough”.*

#### ‘Reassurance for parents about their concerns’

Health professionals also identified that that use of developmental screening tools within a developmental surveillance program was useful in that it allowed practitioners to address parental concerns, which emerged regardless of the parents’ level of education and/or parenting skills,

‘*It’s reassuring then for the parents, ‘The parents want to know that – because sometimes they get people telling them, oh you know, your child might be behind in this or that. Lots of comparison going on and a lot of the times it’s just reassurance for the parents but it’s good to also pick up those children that really do have an issue’(CFHN).*

The provision of information about the child’s developmental progress was perceived to reduce parental anxiety and the opportunity for anticipatory guidance in relation to the next developmental stage.

## Discussion

This paper highlights the perceptions of a purposeful sample of health professionals including CFHNs, GPs and paediatricians regarding the use of developmental screening tools (PEDS and ASQs) as part of a developmental surveillance program in a multicultural and disadvantaged region of NSW, Australia.

‘Professionals’ responses revealed they were aware of the purpose of developmental screening tools such as the PEDS and ASQs. This was particularly the case for CFHNs who used these tools routinely within their professional practice. It was also recognised that the uptake of developmental screening was limited and influenced by a variety of factors. Similar findings of variability in the use of screening tools for developmental surveillance have been reported from Canada and North America [57]. It is known from survey studies that most doctors report using developmental milestone lists or informal checklists as part of an overall strategy of developmental surveillance [58].

The health professionals in our study noted important barriers to developmental surveillance in general, which included difficulties with transportation, and perhaps the infrequent use of translated screening tools for families from CALD backgrounds, where language was raised as being an important barrier. Similar barriers of transport, language and lack of awareness of services for developmental surveillance by parents from CALD backgrounds was also found in another Australian qualitative study [59].

The health professionals in our study noted that parental understanding of expected child development and milestones influences may influence how they respond to PEDS questions. This factor has been studied in a number of contexts with CALD populations. For example, the challenges of using tools such as PEDS with refugee populations from varying ethnic backgrounds are highlighted in a qualitative study from New York, which noted that participants were unsure of a word for “development” in their primary language, demonstrated limited awareness of developmental milestones, and “concern” was unlikely to be raised unless speech or behaviour problems were present [60]. The misinterpretation of the word “concern” in PEDS by the Singaporean Chinese and Malay descent parents as “do you have any feelings or do you care for your child?” without implying any worry about their child’s development has been studied [61]. Immigrant mothers of South American and Japanese descent in North America have also been shown to have limited knowledge of normative child development milestones, as elicited using a validated Knowledge of Infant Development Inventory questionnaire, even after accounting for social-cultural differences in parenting [62]. Despite these challenges PEDS has been validated for different ethnic minorities and with parents from varying levels of education and incomes [63].

The increased acceptance of the PEDS and ASQs by CFHN’s as compared to GPs in our study is not surprising given the fact that it is incorporated as a routine part of training for CFHNs and constitutes a large proportion of their clinical workload, as compared to GPs who manage all health care problems across the life course. Despite GPs recognising the importance of preventive care, well-child care is often an opportunistic activity for them [25]. The findings of challenges in time availability, tool awareness, knowledge of referral pathways and access to services corroborates with previous research that although professionals appreciate the need to routinely do this work they often fail to achieve it. This means that early detection has to include a comprehensive collaborative and integrated process of development promotion, early detection, referral and linkage [64, 65].

GPs in our study highlighted a lack of continuity of care, and the absence of practice nurses as the main barriers in the use of screening tools. They appeared to rely on CFHNs and practice nurses for using screening tools for identifying developmental concerns. The role of practice nurses in the delivery of community child health services in general practice settings is evolving in Australia, in keeping with existing models in the UK, North American and Scandinavian countries [66, 67].More research is needed to better understand acceptability and delivery of developmental screening by practice nurses in Australia.

In the current study, paediatricians (more so than any other profession) highlighted that the availability of intervention services was just as important, if not more important, than a sole focus on screening. Their views that developmental screening activities require more time for consultations, and result in greater referrals to early intervention and allied health services have also been shown in earlier study [40].

### Strengths and limitations

This is a qualitative study in a defined geographical region of Sydney and as such the results cannot be generalised to other parts of Australia or the international context. However, the findings sits within the broad international literature on the challenges in the use of screening tools by health professionals and thus the findings from this study appear to be transferable to other similar settings. Another limitation of the study is that the cultural background of the health professionals and how this impact on their perceptions on the use of screening tools was not explored. Nonetheless, the study has value from gaining the perspectives of GPs, paediatricians and CFHNs regarding barriers for developmental screening that are likely to be relevant to other settings.

We maintained the quality of the study through an awareness of reflexivity and efforts to achieve a high level of interpretive rigour/trustworthiness. This was achieved through feedback from colleagues, checking data analysis with other team members and coding of the analysis by a researcher independent of the data collection, with an acceptable inter-rater reliability. [37]. The other strengths included access to a highly diverse study population, a large cohort of participating health professionals, and the use of both focus groups and individual interviews for data collection. Furthermore, this study is one component of the larger WMG study and supports the preliminary quantitative results of the larger cohort.

### Implications for practice

There are several implications for clinical practice, and policies for child promotion activities. Specifically the following issues need further action:

1. Training and on-going updates in the use of the recommended developmental screening tools are needed particularly for professionals in private practice (GPs and paediatricians). CFHN’s are trained in the use of the tools, and there are clear referral and monitoring pathways. There is an on-going process of updated information for CFHN’s. Moreover, it is also important to emphasise that although relying on the traditional use of developmental milestones can be effective in determining ‘normal’, the concept of 10th to 90th percentile of normative norm is more helpful. The GP college guidelines of a ‘red flag’ approach for identifying developmental concerns are useful in this context [16].
2. There is a need to support GPs, particularly by practice nurses, if enhanced use of development screening tools at general practices is desired in Australia. There is also a need for further discussion about whether the Commonwealth Medicare Benefits Schedules can support GPs in the use of screening tools as part of developmental surveillance. In this context it is important to state that risk assessment screening tools such as the Australian Type 2 Diabetes Risk Assessment Tool (AUSDRISK) for screening are recommended for general practices in Australia within their standard consultations [68].
3. Professionals need a better understanding of factors influencing parents’ perceptions and reports on their child’s development. These issues were further highlighted in our earlier report exploring parents’ perceptions of their health professional which showed that at times the professionals were reluctant to acknowledge parental concerns [22]. CFHNs in NSW receive education about building relationships and improving communication with families. The Family Partnership Model is one approach that has been used, with extensive training for CFHNs. This, or similar training could also be utilised by GPs and paediatricians to consolidate and develop existing skills to better understand parental concerns.
4. There is a need for PEDS trainers to ensure that professionals are provided information about existing translations, and increase their awareness of how to access telephone interpretation services. Considerations for incorporating PEDS scoring guidance and interpretation in the Blue Book should be made, so that the findings from the PEDS are immediately meaningful for GPs and paediatricians. The routine use of PEDS: DM needs further exploration for the NSW health system.
5. Health care professionals particularly the GPs most frequently use mobile reminders and letters for encouraging parents to attend well-visits. There is a need to look at alternate innovative strategies for reminders. In addition, other health promotion and early identification approaches, such as the sustained home nurse visiting programs and other home based vulnerable families’ programs need to be strengthened to engage disadvantaged families.
6. On-going social media community educational approaches for enhancing parental awareness that the well-child visits are "not just about shots", and immunisation visits should also include anticipatory guidance and developmental screening activities.
7. Health system factors particularly the lack of coordination between nursing and GP services, warrant further detailed assessment of the approaches for better integration of care and information sharing between CFHNs and GPs. Some health professional managers have acknowledged that use of a ‘one-stop shop ‘may encourage the increase in use of routine developmental screening in the SW region.

## Conclusion

This study has provided valuable insights into the perceptions and contextual barriers that exist in health care professionals’ participation in the current developmental surveillance program and specifically in the use of screening tools in a multicultural area of relative disadvantage in NSW, Australia.

Further systems and policies research are needed, particularly in relation to the role of practice nurses in the use of developmental screening tools in child health surveillance programs within the context of Australian general practices. Attention is also needed on the use of Commonwealth Medicare Benefits Schedules to support health promotion programs such as developmental surveillance. It is important to address the training needs of professionals in facilitating the use of developmental screening tools, and in promoting the use of these tools for culturally and linguistically diverse populations. A comprehensive and integrated care delivery model with focus on child health promotion and frequent use of educational resources in translated languages in the primary health setting seem to offer a way forward to overcome some of these barriers.

## Abbreviations

AAP: American Academy of Paediatrics
ASQs: Ages and Stages Questionnaires
AUSDRISK: Australian type 2 diabetes risk assessment tool
CALD: Culturally and Linguistically Diverse
CFHN: Child and Family Health Nurses
GPs: General Practitioners
HKC: Healthy Kids Check
NSW: New South Wales
PEDS:DM: Parents Evaluation of Development Status: Developmental Milestones
PEDS: Parents Evaluation of Development Status
PHR: Personal Health Records
SW: South Western Sydney region
SWSLHD: South Western Sydney local health district
UHHV: Universal health home visiting program
UK: The United Kingdom
UNSW: University of New South Wales
